# Theoretical Investigation and Parametric Sensitivity Analysis of Polypropylene–Polyester Fiber-Reinforced Recycled Brick Aggregate Concrete Pavement Humidity Warping Stress During the Service Life

**DOI:** 10.3390/ma18051093

**Published:** 2025-02-28

**Authors:** Fei Li, Shenghao Jin, Peifeng Cheng, Zehui Wang

**Affiliations:** 1 Changda Construction Technology Co., Ltd., Weifang 261205, China; xiaofei2724@163.com; 2 School of Civil Engineering and Transportation, Northeast Forestry University, Harbin 150040, China; shenghao.jin@nefu.edu.cn (S.J.); 13516349060@163.com (Z.W.)

**Keywords:** recycled brick aggregate, polypropylene fiber, PPRBAC pavement, humidity warping, orthogonal experiment, gray correlation analysis

## Abstract

Pavement humidity warping is a critical factor limiting the application of PPRBAC on low-volume roads. A nonlinear wet-warping stress formula for PPRBAC slabs has been derived based on previous experimental results, and the finite element method was employed to develop a single-board model in order to verify the accuracy of the analytical solution. Subsequently, the finite difference method, in conjunction with the finite element method, was employed to investigate the calculation methodology for wet-warping stress in PPRBAC slabs during service. Finally, the Taguchi–GRA (gray relational analysis) method was selected to analyze the sensitivity of humidity warping factors affecting PPRBAC slabs. The findings indicate that compared to the traditional bending moment equivalent method, the wetness warping stress formula established in this study accounts for the nonlinearity of wetness warping stress and demonstrates higher accuracy. For PPRBAC pavements during the service period, assuming uniform initial humidity distribution along the height within the concrete does not align with practical observations. The calculated humidity warping stress and deformation using this assumption are 1.1 and 1.7 times those obtained from the comprehensive dry–wet calculation method. It is crucial to consider the wet stage’s impact on the dry stage in the calculations. The Taguchi–GRA method objectively determines the weight of factors affecting humidity warping in PPRBAC, with slab size, thickness, and flexural strength having the greatest influence.

## 1. Introduction

In recent years, the global emphasis on green, low-carbon, energy-saving, and environmental protection has emerged as a defining theme of our time. As traditional high-energy-consuming sectors, the construction and transportation infrastructure industries significantly contribute to carbon emissions. Consequently, these industries’ energy conservation and emission reduction efforts have increasingly focused on using green building materials. After dismantling, construction waste is processed through crushing, screening, and cleaning to produce recycled aggregate. The resulting recycled concrete represents an essential category of green building materials today. Depending on the aggregate source, recycled concrete can be classified into Recycled Concrete Aggregate Concrete (RCAC) and Recycled Brick Aggregate Concrete (RBAC). Compared with RCAC, RBAC exhibits disadvantages such as lower bulk density, crushing index, and water absorption rate, significantly limiting its mechanical properties [1,2,3].

Extensive research has been conducted on the mechanical properties of Recycled Brick Aggregate Concrete (RBAC). Wu et al. [4] investigated the impact of Recycled Brick Aggregate (RBA) on the mechanical properties of RBAC under various drying methods. Air-dried RBAC exhibited superior mechanical performance. Meng et al. [5] suggested that RBA significantly influences RBAC’s mechanical properties, which become more pronounced as the RBA substitution rate increases. Tan et al. [6] examined the impact of the RBA substitution rate on RBAC’s mechanical properties and freeze–thaw resistance from macroscopic and microscopic perspectives, elucidating the damage mechanisms during freeze–thaw cycles. These studies collectively indicate that an increase in the RBA substitution rate generally leads to a decline in RBAC’s durability and mechanical properties. To enhance the mechanical properties of RBAC, incorporating fibers is a conventional method that can mitigate crack formation through their bridging effect.

Commonly used fiber types include steel fiber [7,8,9], polypropylene fiber [10], and glass fiber [11]. Polypropylene–polyester fiber (PPF) is particularly suitable for RBAC due to its excellent tensile strength, cost-effectiveness, and resistance to acids and alkalis. However, there is currently no standardized optimal dosage range for PPF. Mak et al. [12] found that a 1% PPF dosage yielded the best toughening effect. Jorbat et al. [13] demonstrated that a 0.35% PPF dosage was optimal for improving the mechanical properties of two types of ordinary concrete. Although the optimal dosage ranges vary, the observed trends are consistent: PPF enhances the flexural strength of RBAC more effectively than its compressive strength. Consequently, PPRBAC (PPF-Reinforced Recycled Brick Aggregate Concrete) is better suited for flexural components such as road surfaces than compression components. Moreover, Li et al. [14] utilized lifecycle assessments to demonstrate that the excessive use of PPF could reduce environmental benefits, leading to limitations in terms of PPF content and a constrained enhancement of flexural strength. In summary, considering the relatively limited strength of PPRBAC, its most effective and sustainable application is as a low-grade pavement material.

For cement pavement structures, the influence of humidity gradients is particularly significant. Compared to temperature gradients, humidity gradients exhibit prolonged duration and induce more significant curing stresses [15,16], posing a formidable challenge for PPRBAC pavements. Wei et al. [17] conducted concrete moisture diffusion tests and discovered that, under extreme conditions, the linear temperature gradient equivalent to the humidity gradient could reach 107 °C/m, significantly exceeding the maximum temperature gradient specified by codes [18]. This finding underscores the non-negligible warping effects induced by humidity. Wang et al. [19] derived an equivalent analytical formula for humidity in non-uniform free slabs, incorporating temperature gradients, and validated this formula through comparisons between the Westergaard method and finite element solutions, thereby analyzing reinforcement sensitivity factors. Zhu et al. [20] investigated the impact of various road-like parameters on the stress and deformation of cement pavements. Chen et al. [21] examined the stress development in unloaded cement pavements under the combined effects of axial load, temperature, and water, elucidating the relationships between these factors and stress. These studies provide a comprehensive foundation for theoretical research on calculating warping stresses in PPRBAC pavement slabs under nonlinear humidity gradients and conducting parameter sensitivity analyses. However, the analysis process in some studies may be overly subjective.

Given the current challenges, including an imperfect theoretical framework for calculating humidity warpage stress in PPRBAC and the subjective sensitivity analysis of influencing factors, a nonlinear wet-warping stress formula for PPRBAC based on flexural strength is derived, leveraging previous experimental results. This study emphasizes the calculation method of wet-warping stress for in-service PPRBAC using the FDM (finite difference method)–FEM (finite element method). Furthermore, to address biases introduced by subjective factors in the sensitivity analysis, the Taguchi–GRA (gray correlation analysis) method is employed to determine each influencing factor’s weight comprehensively. This research enhances the calculation method for humidity warpage in PPRBAC pavements during their service life, providing a more robust theoretical foundation for the application of PPRBAC in low-volume roads.

## 2. Theoretical Methods

### 2.1. Elastic Modulus Prediction Model of PPRBAC

**Figure 1:**
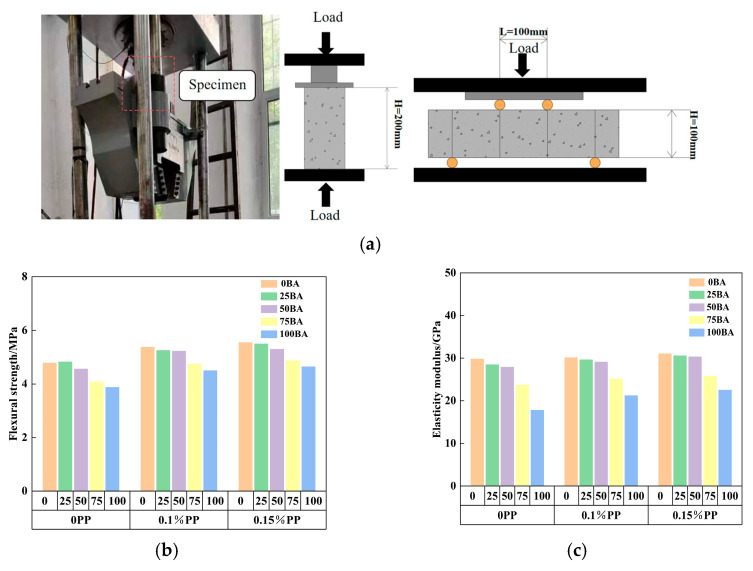
The test results of the mechanical properties of PPRBAC: (**a**) sample and experimental equipment; (**b**) flexural strength results; (**c**) elastic modulus results (experimental data from previous research results [14]).

The elastic modulus has a significant effect on the warping stress of PPRBAC slab, and its accurate estimation is key to improving the calculation accuracy of humidity-induced warping stress. In addition, in pavement applications, flexural strength is usually used as a control index. Establishing a conversion relationship between bending strength and the elastic modulus can lead to a more valuable analytical solution for the humidity-induced warping stress of PPRBAC, expressed in terms of bending strength. In order to establish the corresponding elastic modulus prediction model, this study further explores the conversion relationship between the flexure strength of PPRBAC and the elastic modulus on the basis of our previous research group. Sample preparation and detailed test procedures can be found in reference [14] (see Figure 1a for test equipment and sample size). Figure 1b,c show the experimental results of elastic modulus and bending strength used to build the prediction model.

**Figure 2:**
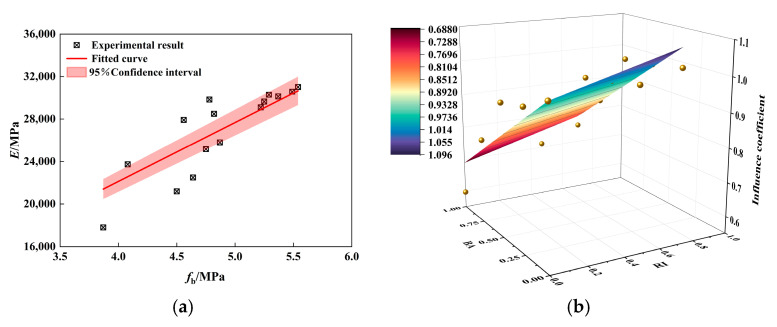
PPRBAC elastic modulus prediction model: (**a**) the relationship between flexural strength and elastic modulus; (**b**) correction factor.

The BA and PP content substitution rate was considered to establish the conversion relationship between flexural strength and elastic modulus [14]. Firstly, a linear regression analysis was conducted on the relationship between flexural strength and elastic modulus (Figure 2a), with the regression results presented in Equation (1). A strong linear relationship exists between the flexural strength *f_c_* and the elastic modulus *E*, with an R^2^ value of 0.99, indicating a high degree of correlation. Secondly, the ratio of each set of data to ordinary concrete was defined as the weakening effect of BA and the toughening effect of PP, using the ξ expression. The toughening effect of PPF on PPRBAC was defined by the fiber toughening coefficient $\mathrm{RI}=V_{\mathrm{PPF}}\times l_{\mathrm{PPF}}/d_{\mathrm{PPF}}$. By comprehensively considering the weakening effect of BA and the toughening effect of PPF, Equation (2) can be derived. The regression correlation coefficient for this equation is R^2^ = 0.85 (see Figure 2b). Consequently, the conversion relationship between the flexural strength and elastic modulus (Equation (3)) can be established by combining Equations (1) and (2).

(1) $$E=5534.1f_{b}$$

(2) ξ=1+0.135RI−0.310BA

(3) $$E=5534.1\left(1+0.135\mathrm{RI}-0.310\mathrm{BA}\right)f_{b}$$

### 2.2. Calculation Method for Nonlinear Humidity Warping of PPFRAC Slabs

The PPRBAC pavement is assumed to remain in continuous contact with the substructure, with no gaps present. Additionally, the pavement’s self-weight is neglected, and it is considered an elastic thin slab. Based on the elastic thin slab hypothesis and considering the influence of humidity on PPRBAC, the stress–strain relationship can be expressed as Equations (4) and (5). Here, $\varepsilon_{x}$ and $\varepsilon_{y}$ represent the strain components in the *x* and *y* directions, respectively, while $\sigma_{x}$ and $\sigma_{y}$ denote the stress components in the *x* and *y* directions. *E* is the elastic modulus of PPRBAC, *v* is Poisson’s ratio, $\alpha_{H}$ is the coefficient of moisture growth, and $H\left(z\right)$ is the humidity distributed along the depth of the slab, ranging from 0 to 1.

(4) $$\varepsilon_{x}=\left(\sigma_{x}-\nu \sigma_{y}\right)/E+\alpha_{H}H\left(z\right)$$

(5) $$\varepsilon_{y}=\left(\sigma_{y}-\nu \sigma_{x}\right)/E+\alpha_{H}H\left(z\right)$$

The coefficient of wet expansion in the equation can be determined using Equation (6) [16], where $\alpha_{H}$ quantifies the humidity-induced shrinkage deformation of PPRBAC and *H* indicates the relative humidity. The value ranges from 0 to 1.

(6) $$\alpha_{H}=\frac{1}{H}6150\times 10^{-6}\times \left(1-H\right)\times {\left(1-V_{A}\right)}^{n}$$

At this juncture, by incorporating Equations (4) and (6), the stress components can be formulated as follows:

(7) $$\sigma_{x}=5534.1\left(1+0.135\mathrm{RI}-0.310\mathrm{BA}\right)\times \left[\frac{\varepsilon_{x}+\nu \varepsilon_{y}}{1-v^{2}}-\frac{\alpha_{H}H\left(z\right)}{1-v}\right]f_{b}$$

(8) $$\sigma_{y}=5534.1\left(1+0.135\mathrm{RI}-0.310\mathrm{BA}\right)\times \left[\frac{\varepsilon_{y}+\nu \varepsilon_{x}}{1-v^{2}}-\frac{\alpha_{H}H\left(z\right)}{1-v}\right]f_{b}$$

The road slab is assumed to behave like a thin elastic slab based on the theory of thin elastic slabs. By incorporating the geometric Equations (9) and (10), Equations (7) and (8) can be reformulated as Equations (11) and (12). In these equations, ω denotes the deflection of the PPRBAC slab.

(9) $$\varepsilon_{x}=\frac{\partial u}{\partial x}=-\frac{\partial^{2}\omega }{\partial x^{2}}z$$

(10) $$\varepsilon_{y}=\frac{\partial \nu }{\partial x}=-\frac{\partial^{2}\omega }{\partial y^{2}}z$$

(11) $$\sigma_{x}=5534.1\left(1+0.135\mathrm{RI}-0.310\mathrm{BA}\right)\times \left[-\frac{z}{1-\nu^{2}}\left(\frac{\partial^{2}\omega }{\partial x^{2}}+\nu \frac{\partial^{2}\omega }{\partial y^{2}}\right)-\frac{1}{1-\nu }\alpha_{H}H\left(z\right)\right]f_{b}$$

(12) $$\sigma_{y}=5534.1\left(1+0.135\mathrm{RI}-0.310\mathrm{BA}\right)\times \left[-\frac{z}{1-\nu^{2}}\left(\frac{\partial^{2}\omega }{\partial y^{2}}+\nu \frac{\partial^{2}\omega }{\partial x^{2}}\right)-\frac{1}{1-\nu }\alpha_{H}H\left(z\right)\right]f_{b}$$

Based on Equations (11) and (12), integrating the PPRBAC along the thickness direction yields the bending moments in the *x* and *y* directions under a linear moisture gradient, as given by Equations (13) and (14).

(13) $$M_{x}=-D\left(\frac{\partial^{2}\omega }{\partial x^{2}}+\nu \frac{\partial^{2}\omega }{\partial y^{2}}\right)+M_{H}$$

(14) $$M_{y}=-D\left(\frac{\partial^{2}\omega }{\partial y^{2}}+\nu \frac{\partial^{2}\omega }{\partial x^{2}}\right)+M_{H}$$

In Equations (13) and (14), the terms *D* and *M_H_* can be reformulated as presented in Equations (15) and (16).

(15) $$D=-\frac{461.2z^{3}}{1-\nu^{2}}\left(1+0.135\mathrm{RI}-0.310\mathrm{BA}\right)$$

(16) $$M_{H}=\frac{5534.1\left(1+0.135\mathrm{RI}-0.310\mathrm{BA}\right)f_{c}}{1-\nu }\int_{-0.5\;z}^{0.5\;z}\alpha_{H}H\left(z\right)zdz$$

At this juncture, the calculation method proposed in [22] is employed, wherein the temperature-induced moment is equated to the moisture-induced moment. Consequently, *M_H_* can be expressed as Equation (17). In this equation, *α* denotes the linear thermal expansion coefficient, and $T\left(z\right)$ represents the temperature gradient distributed along the height of the slab.

(17) $$M_{H}=M_{T}=\frac{5534.1\left(1+0.135\mathrm{RI}-0.310\mathrm{BA}\right)f_{c}}{1-\nu }\int_{-0.5\;z}^{0.5\;z}\alpha_{T}T\left(z\right)zdz$$

If the PPRBAC slab was assumed to be of infinite dimensions, its deflection could be approximated as zero. In this case,

(18) $$M_{x}=M_{y}=M_{H}=\frac{5534.1\left(1+0.135\mathrm{RI}-0.310\mathrm{BA}\right)f_{c}}{1-\nu }\int_{-0.5\;z}^{0.5\;z}\alpha_{H}H\left(z\right)zdz$$

When both the length and width of the PPRBAC slab are finite, the analysis can draw upon the methodology for temperature-induced warping stress. Assuming that the slab length is *a* and the width is *b*, the deflection of the slab can be described by Equation (19). In the formula, the deflection of the PPRBAC slab with finite length a and infinite width b is $\omega \left(x\right)$, and the deflection of the PPRBAC slab with finite length b and infinite width a is $\omega \left(y\right)$.

(19) $$\omega \left(x,y\right)=\omega \left(x\right)+\omega \left(y\right)$$

At this point, the maximum moisture warping stress can be further expressed as Equations (20) and (21).

(20) $$\sigma_{x}=\frac{2767M_{H}z}{\left(1-\nu^{2}\right)\left(1+\nu \right)D}\left(C_{x}+\nu C_{y}\right)\left(1+0.135\mathrm{RI}-0.310\mathrm{BA}\right)f_{b}$$

(21) $$\sigma_{x}=\frac{2767M_{H}z}{\left(1-\nu^{2}\right)\left(1+\nu \right)D}\left(C_{y}+\nu C_{x}\right)\left(1+0.135\mathrm{RI}-0.310\mathrm{BA}\right)f_{b}$$

In the aforementioned equations, *C_x_* and *C_y_* denote the coefficients for calculating the warping stress, which are summarized in Equations (22) and (23). In these equations, a and b represent the length and width of the PPRBAC slab, respectively, as previously discussed. *L* denotes the stiffness radius, which is expressed by Equation (24), where *K* represents the subgrade reaction modulus, measured in MPa.

(22) $$\begin{array}{l} C_{x}=1-\frac{2\cos\left(a/2\sqrt{2}l\right)\cos\left(za/2\sqrt{2}l\right)}{\sin\left(a/\sqrt{2}l\right)+\sin\left(za/\sqrt{2}l\right)}\times \left[\left(\tan\left(a/2\sqrt{2}l\right)+\tan\left(za/2\sqrt{2}l\right)\right)\right.\\ \left.\cos\left(x/\sqrt{2}l\right)\cos\left(zx/\sqrt{2}l\right)\right]+\left[\left(\tan\left(a/2\sqrt{2}l\right)-\tan\left(za/2\sqrt{2}l\right)\right)\sin\left(x/\sqrt{2}l\right)\sin\left(zx/\sqrt{2}l\right)\right] \end{array}$$

(23) $$\begin{array}{l} C_{y}=1-\frac{2\cos\left(b/2\sqrt{2}l\right)\cos\left(zb/2\sqrt{2}l\right)}{\sin\left(b/\sqrt{2}l\right)+\sin\left(zb/\sqrt{2}l\right)}\times \left[\left(\tan\left(b/2\sqrt{2}l\right)+\tan\left(zb/2\sqrt{2}l\right)\right)\right.\\ \left.\cos\left(y/\sqrt{2}l\right)\cos\left(zy/\sqrt{2}l\right)\right]+\left[\left(\tan\left(b/2\sqrt{2}l\right)-\tan\left(zb/2\sqrt{2}l\right)\right)\sin\left(y/\sqrt{2}l\right)\sin\left(zy/\sqrt{2}l\right)\right] \end{array}$$

(24) $$l=4\sqrt{461.2\left(1+0.135\mathrm{RI}-0.310\mathrm{BA}\right)f_{c}z^{3}/\left(1-\nu^{2}\right)K}=4\sqrt{D/K}$$

**Figure 3:**
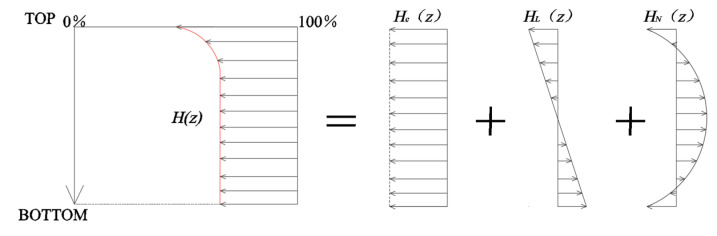
Diagram of the decomposition process of humidity gradient.

According to the literature [22], the humidity gradient *H*(*z*) can be decomposed into the sum of three distinct components: *H_c_*(*z*), representing the strain due to constant humidity; *H_L_*(*z*), representing the strain induced by a linear humidity gradient; and *H_N_*(*z*), representing the strain caused by a nonlinear humidity gradient. The decomposition process is illustrated in Figure 3.

Specifically, *H_c_*(z) signifies the uniform contraction of the slab, which can be assumed to produce no stress in the absence of lateral constraints. *H_L_*(z) induces warping deformation and can be analyzed using the methodology outlined in Section 2.2. *H_N_*(z) does not cause either warping deformation or uniform contraction but results in internal strain, leading to self-balancing stresses. Therefore, when calculating the warping stress under a nonlinear humidity gradient, the stress distribution should be computed separately for each decomposed component and then superimposed to obtain the overall stress distribution.

Since the nonlinear component *H_N_*(*z*) does not warp, then the deflection *w* of the slab is 0, and then the warp stress caused by the nonlinear humidity gradient can be obtained from Equations (11) and (12) (Equation (25)):

(25) $$\sigma_{Nx}\left(z\right)=\sigma_{Ny}\left(z\right)=-\frac{5534.1\left(1+0.135\mathrm{RI}-0.310\mathrm{BA}\right)f_{c}}{1-\nu }\alpha_{H}H_{N}\left(z\right)$$

By converting Equation (25) into the corresponding temperature gradient based on the principle of contraction equivalence (Equation (26)), the resulting temperature gradient can be decomposed into three components: *T_c_*(*z*), representing the strain due to constant conditions; *T_L_*(*z*), representing the strain due to linear conditions; and *T_N_*(*z*), representing the strain due to nonlinear conditions. The components *T_c_*(*z*) and *T_L_*(*z*) can be calculated using Equations (27) and (28). Here, *T*_0_ denotes the reference temperature, and its specific value does not influence the calculation results.

(26) $$\Delta T=-\frac{1}{\alpha }6150\times 10^{-6}\times \left(1-H\right)\times {\left(1-V_{A}\right)}^{n}$$

(27) $$T_{c}\left(z\right)=T_{0}+\frac{\int_{h}\alpha \left(z\right)5534.1\left(1+0.135\mathrm{RI}-0.310\mathrm{BA}\right)f_{b}(z)\left[T_{z}-T_{0}\right]dz}{\alpha \left(z\right)\int_{h}5534.1\left(1+0.135\mathrm{RI}-0.310\mathrm{BA}\right)f_{b}(z)dz}$$

(28) $$T_{L}\left(z\right)=T_{0}+\frac{z\int_{h}\alpha \left(z\right)5534.1\left(1+0.135\mathrm{RI}-0.310\mathrm{BA}\right)f_{b}(z)\left[T_{z}-T_{0}\right]zdz}{\alpha \left(z\right)\int_{h}5534.1\left(1+0.135\mathrm{RI}-0.310\mathrm{BA}\right)f_{b}(z)z^{2}dz}$$

The classification of nonlinear strain must satisfy the premise outlined in Equation (29); consequently, the nonlinear strain component can be expressed by Equation (30).

(29) $$T\left(z\right)-T_{0}=\left[T_{c}\left(z\right)-T_{0}\right]+\left[T_{L}\left(z\right)-T_{0}\right]+\left[T_{N}\left(z\right)-T_{0}\right]$$

(30) $$T_{N}\left(z\right)=T\left(z\right)-T_{c}\left(z\right)-T_{L}\left(z\right)+2T_{0}$$

At this point, we assume that the PPRBAC slab is homogeneous, meaning that the flexural rigidity (*f_b_*) and coefficient of linear expansion α are constant throughout the height of the slab and do not vary with depth (*z*). Consequently, *T_c_*(*z*), *T_L_*(z), and *T_N_*(*z*) can be expressed as Equations (31)–(33), respectively.

(31) $$T_{c}(z)=\frac{1}{h}\int_{-0.5\;h}^{0.5\;h}T(z)dz$$

(32) $$T_{L}\left(z\right)=T_{0}+\frac{12\;z}{h^{3}}\int_{-0.5\;h}^{0.5\;h}T\left(z\right)zdz$$

(33) $$T_{N}(z)=T_{0}+T(z)-\frac{1}{h}\int_{-0.5\;h}^{0.5\;h}T(z)dz-\frac{12\;z}{h^{3}}\int_{-0.5\;h}^{0.5\;h}T(z)zdz$$

Based on Equation (25), the internal stress can be reformulated as follows:

(34) $$\sigma_{Nx}\left(z\right)=\sigma_{Ny}\left(z\right)=-\frac{5534.1\left(1+0.135\mathrm{RI}-0.310\mathrm{BA}\right)f_{c}}{1-\nu }\alpha_{H}\left[T_{N}\left(z\right)-T_{0}\right]$$

The analytical solution of nonlinear humidity warpage stress for a specific type of fiber-reinforced pavement (PPRBAC pavement) is derived. For other types of fiber-reinforced recycled concrete pavement, the relation of laboratory experimental mechanical parameters can be re-established, and the corresponding analytical solutions can be obtained by referring to the calculation process in this paper.

### 2.3. Gray Relational Computing Theory

To analyze the parameter sensitivity of the humidity-induced warping stress in PPRBAC pavements, the Taguchi method in conjunction with gray correlation analysis is employed. The foundation of the GRA theory is the linear interpolation method, which is an analytical method for finding the degree of correlation between the main behavior factor and the behavior factors. This method geometrically processes the data and calculates the degree of correlation between the behavior factors and the main behavior factor based on theoretical formulas. The moisture warping stress of PPRBAC pavement is the result of multi-factor nonlinear coupling. The gray relational theory has an absolute advantage for sparse and few data. It can be used to determine the weights in the comprehensive evaluation of the system and the similarity between the known system and the unknown system [23,24,25,26]. Therefore, the gray relational theory is one of the best methods for analyzing the sensitivity of various factors in moisture warping.

In this analysis, the primary behavioral factors, namely maximum humidity-induced warping stress and displacement, are designated as the reference series (Equation (35)). The influencing factors are designated as the comparison series (Equation (36)). Herein, *k* represents the number of groups in the orthogonal experiments, and i denotes the number of behavioral factors.

(35) $$Xo(k)=\left[Xo(1),Xo(2),\cdots ,\;Xo(n)\right]$$

(36) $$Xi(k)=\left[Xi(1),Xi(2),\cdots ,\;Xi(n)\right]$$

The main behavioral factor either increases or decreases as the behavioral factor increases. Data can be rendered dimensionless using Equation (37). The correlation coefficient between the main behavioral factor and the influencing factors is expressed by Equation (38). In these equations, $\alpha =\min\left|W0(k)-Wi(k)\right|$, $\beta =\max\left|W0(k)-Wi(k)\right|$. The identification coefficient ρ is typically set to 0.5. The correlation degree of behavioral factors can be described using Equation (39).

(37) $$\left\{\begin{array}{c} Wi(k)=\left[1,Xi(2)/Xi(1),\cdots ,Xi(n)/Xi(1)\right]\\ Wi(k)=\left[1,Xi(1)/Xi(2),\cdots ,Xi(1)/Xi(n)\right] \end{array}\right.$$

(38) $$\xi i\left(k\right)=\frac{\min\alpha +\rho \max\beta }{\Delta i(k)+\rho \max\beta }$$

(39) $$\gamma i=\frac{1}{n}\sum_{i=1}^{n}\xi i(k)$$

## 3. Results and Discussion

### 3.1. PPRBAC Pavement Humidity Warping Stress Theoretical Analytical Method

The capillary water absorption and self-drying efficiency of cement-based materials are primarily influenced by their pore structure and the external environment. Consequently, the internal humidity transfer mechanism of PPRBAC is analogous to that of conventional concrete. To validate the accuracy of the humidity warping stress calculation theory for PPRBAC proposed in Section 2, we utilized the concrete moisture transfer experiment described in the literature [27] as a benchmark. Equation (40) represents the nonlinear temperature gradient equivalent to the relative humidity derived from this experiment.

(40) $$T\left(z\right)=0.8742+12.7332\;z+101.0502\;z^{2}-4198.2076\;z^{3}-37104.6468\;z^{4}$$

When the equivalent temperature gradient is represented as a quartic polynomial, as given in Equation (41), the corresponding components can be expressed through Equations (42)–(44) by substituting the expressions from Equations (31)–(33).

(41) $$T\left(z\right)=c+dz+ez^{2}+fz^{3}+gz^{4}$$

(42) $$T_{c}\left(z\right)=c+\frac{e}{12}h^{2}+69.2gh^{4}$$

(43) $$T_{L}\left(z\right)=T_{0}\left(d+\frac{3}{20}fh^{2}\right)z$$

(44) $$T_{N}\left(z\right)=T_{0}-\frac{e}{12}h^{2}-69.2gh^{4}-\frac{3f}{20}h^{2}z+ez^{2}+fz^{3}+gz^{4}$$

**Figure 4:**
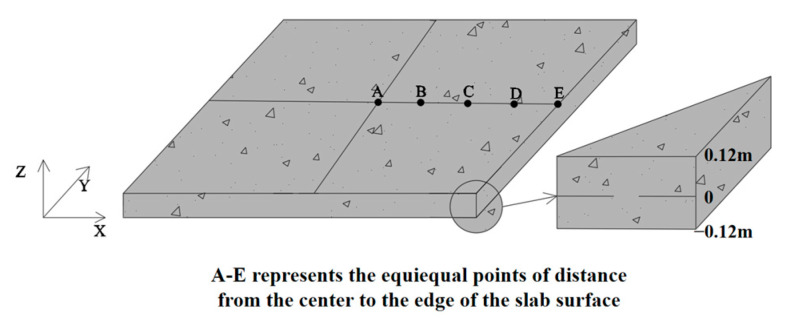
PPRBAC slab humidity warping stress calculation point.

In the calculation process, the flexural strength of the PPRBAC slab was determined based on prior research findings [14], selecting the configuration that offers the optimal balance between economic and environmental benefits. It corresponds to a BA substitution rate of 50% and a PPF content of 0.1%. The material’s Poisson’s ratio was set to 0.18, the aggregate volume fraction to 0.75, and the slab dimensions were specified as 4 m × 4 m × 0.24 m. The subgrade reaction modulus was selected as 110 MPa. This study focused on a PPRBAC slab with four free edges, and the stress induced by *T_c_*(*z*) was neglected in the analysis. Given the square geometry of the slab, which exhibits central symmetry, the calculations were conducted at five equidistant points on the slab surface (As shown in Figure 4).

**Figure 5:**
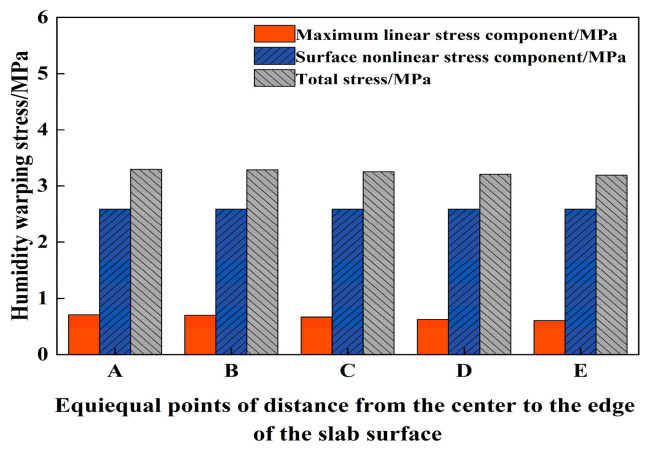
Analytical solution of nonlinear moisture warping stress.

The corresponding linear stress component is obtained by substituting Equation (40) into Equation (43). This component can then be substituted into Equation (17) to derive the equivalent moment, thereby enabling the calculation of warping stresses at different surface locations of the PPRBAC. Substituting Equation (40) into Equation (44) yields the nonlinear stress component, which can subsequently be substituted into Equation (34) to determine the self-balancing stresses at various depths, with the maximum value occurring at the surface of the slab. Given the assumption that the slab has free edges, the linear and nonlinear stress components generated on the surface of the PPRBAC slab can be superimposed to calculate the total stress at the center of the top. The results of these calculations are presented in Figure 5, indicating that the warping stress is most significant in the center of the slab surface.

To facilitate a more comprehensive comparison between the current method and existing approaches, the bending moment equivalent method is employed for this analysis. The bending moment induced by the humidity gradient is presented in Equation (45):

(45) $$M_{m}=\int_{-0.5\;h}^{0.5\;h}\sigma \left(z\right)zdz=\frac{5534.1\left(1+0.135\mathrm{RI}-0.310\mathrm{BA}\right)f_{b}(z)}{1-\nu }\int_{-0.5\;h}^{0.5\;h}\varepsilon_{H}\left(z\right)zdz$$

Among these factors, the relationship between shrinkage deformation and internal humidity can be described by the Pickett constitutive relationship [22].

(46) $$\varepsilon_{H}=\varepsilon_{p}\times {\left(1-V_{A}\right)}^{n}=6150\times 10^{-6}\times \left(1-H\right)\times {\left(1-V_{A}\right)}^{n}$$

In this context, the bending moment resulting from a linear temperature gradient can be articulated as follows:

(47) $$M_{T}=\frac{461.2\left(1+0.135\mathrm{RI}-0.310\mathrm{BA}\right)f_{b}\Delta T_{e}\alpha h^{2}}{1-\nu }$$

At this stage, the bending moment induced by humidity is equivalent to that caused by temperature. Consequently, the linear temperature gradient under these equivalent conditions can be derived $\Delta T_{L}$ (see Equation (48)).

(48) $$\Delta T_{L}=\frac{12}{\alpha h^{2}}\int_{-0.5\;h}^{0.5\;h}\varepsilon_{c}\left(z\right)zdz$$

**Figure 6:**
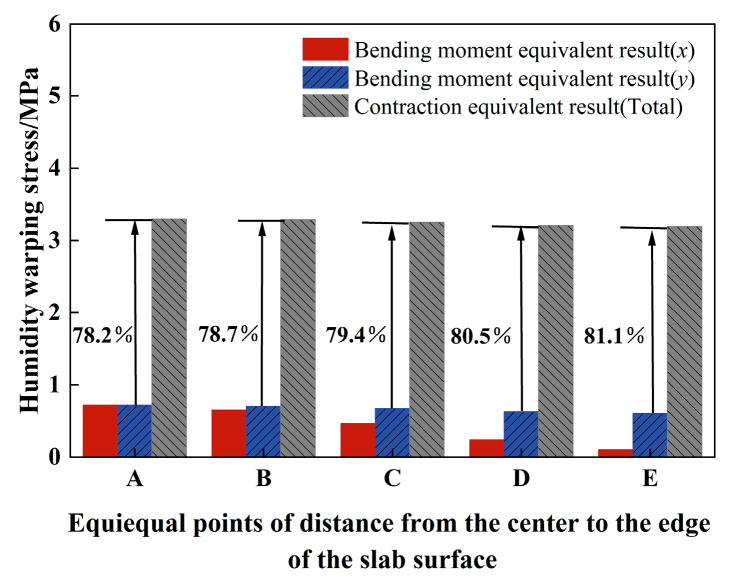
Comparison of calculation results of different equivalent methods.

Furthermore, the stress is computed in the *x* and *y* directions at five distinct locations on the slab’s surface, and these values are compared with the total stress presented in Figure 5 (refer to Figure 6). It is evident that the stress resulting from the equivalent linear temperature gradient, as calculated using the bending moment equivalence method, is significantly lower than the nonlinear stress obtained through the contraction equivalence method. However, when compared to the $\max\left(\sigma_{x},\sigma_{y}\right)$ and linear stress component depicted in Figure 5, they exhibit a high degree of similarity. The bending moment equivalent method primarily focuses on the deformation induced by the *H_L_*(z) component and performs an equivalence transformation. This approach, however, overlooks the self-equilibrating stress generated by the PPRBAC slab under a humidity gradient. In contrast, the shrinkage equivalent method directly accounts for the total deformation, fully considering the self-equilibrating stress arising from the nonlinear *H_N_*(z) component. Consequently, this method effectively captures the nonlinearity inherent in the warping deformation process. The maximum tensile stress under the two hypotheses is further analyzed (position A). The analytical solution of the maximum stress derived from the bending moment method constitutes 21.8% of the shrinkage equivalent value. Under this assumption, the pavement will not crack or fail due to the humidity gradient effect. However, the results obtained from the shrinkage equivalent assumption have exceeded the fatigue strength of the selected mix ratio in this study (as per literature [28], the maximum flexural strength [14] is 0.6, i.e., 3.132 MPa). This clearly indicates that the nonlinear humidity component cannot be neglected.

### 3.2. Reliability Verification of Shrinkage Equivalent Calculation Method

To further validate the reliability of the method proposed in this paper, a finite element method is employed to corroborate the analytical solution. As previously mentioned, a PPRBAC pavement panel measuring 4 m × 4 m has been established, with an expanded foundation to minimize boundary effects. The parameters for the surface layer remain consistent with those outlined in Section 3.1. A subgrade is constructed on a Winkler foundation, featuring a thickness of 0.3 m, an elastic modulus of 1500 MPa, a Poisson’s ratio of 0.2, and a 2400 kg/m³ material density.

**Figure 7:**
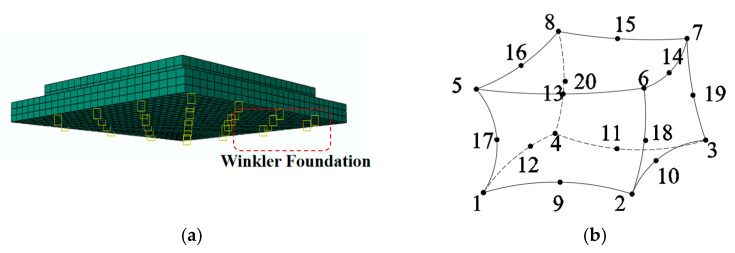
Model construction and grid partitioning: (**a**) finite element model; (**b**) C3D20 element.

The surface layer and subgrade are assumed to be continuous to ensure consistency with prior theoretical calculations (the two layers are connected using tie elements), neglecting the surface layer’s self-weight. The model’s mesh division and element selection are illustrated in Figure 7; specifically, the C3D20 element (shown in Figure 7b) employs a quadratic formulation to describe the displacement field. For analyzing warping displacements within the PPRBAC slab, actual boundary conditions approximate a quadratic curve. In this context, utilizing this element for mesh division offers significant advantages when computing panel bending and ensures high calculation accuracy even with a limited number of mesh elements.

**Figure 8:**
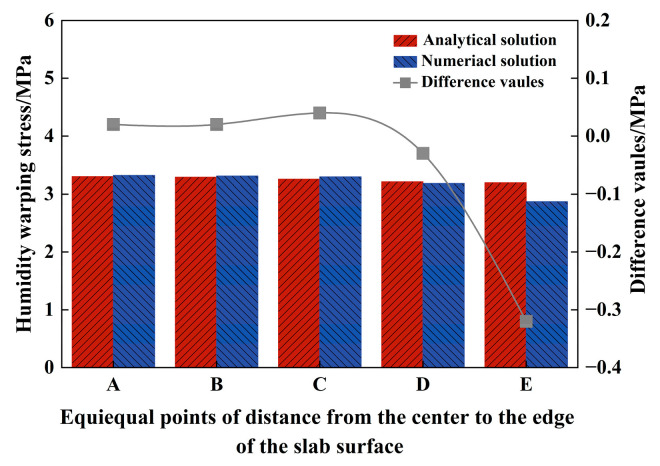
Comparison of numerical results with analytical solutions.

Figure 8 illustrates the computed slab surface stress following extraction. By comparing the extracted finite element analysis results with the analytical solution of nonlinear humidity-induced warping stress, it is evident that the proposed PPRBAC warping stress analytical solution aligns well with the numerical solution, demonstrating an overall average relative error of merely 2.23%. Apart from location E, which exhibits discrepancies due to finite element boundary effects (at this position, the maximum relative error reached 10.02%), the numerical outcomes at the remaining four locations are virtually identical to the analytical solution. This corroborates the accuracy and validity of the theoretical findings presented.

### 3.3. Calculation of PPRBAC Pavement Moisture Warping Stress During Service Period

The humidity gradient within PPRBAC was simulated before calculating the humidity-induced warping stress. Two working conditions, namely the initial pouring stage and the service period, were selected for analysis. External drying and capillary water absorption of cement-based materials significantly contribute to moisture-induced warping [29]. In this study, relative humidity H was chosen as a variable to represent the internal moisture content of PPRBAC. The one-dimensional diffusion equation (Equation (49)) can describe the internal moisture at the initial pouring stage. For PPRBAC during the service period, since the hydration process has been completed, the effect of self-drying is neglected, which is expressed by Equation (50). Here, D represents the humidity diffusion coefficient, and Hs denotes the humidity change due to material drying.

(49) $$\frac{\partial H}{\partial t}=\frac{\partial }{\partial x}\left(D\left(H\right)\frac{\partial H}{\partial x}\right)+\frac{\partial H_{s}}{\partial t}$$

(50) $$\frac{\partial H}{\partial t}=\frac{\partial }{\partial x}\left(D\left(H\right)\frac{\partial H}{\partial x}\right)$$

The same governing equation and different diffusion coefficients are used for the drying process and wetting process. The diffusion coefficient of the drying process is expressed in Equation (51), while that of the wetting process is expressed in Equation (52). In the formula, *D*_0_ is the diffusion coefficient under the saturation state of the material. *α*, *H_c_*, and *n* are regression parameters, and the values are related to the drying boundary. The drying boundary and parameters used in this paper are the same as those in the literature [29]. m is the empirical value of 6 (according to references [29,30], this value has a high reliability through laboratory experiments and genetic algorithm verification). *D*_1_ is the diffusion coefficient under the dry state of the material. The BET model is used to realize the conversion between saturation θ and relative humidity *H* [30].

(51) $$D\left(H\right)=\alpha D_{0}+\left(\alpha +\frac{1-\alpha }{1+{\left(\frac{1-H}{1-H_{c}}\right)}^{n}}\right)$$

(52) $$D\left(\theta \right)=D_{1}e^{m\theta }$$

**Figure 9:**
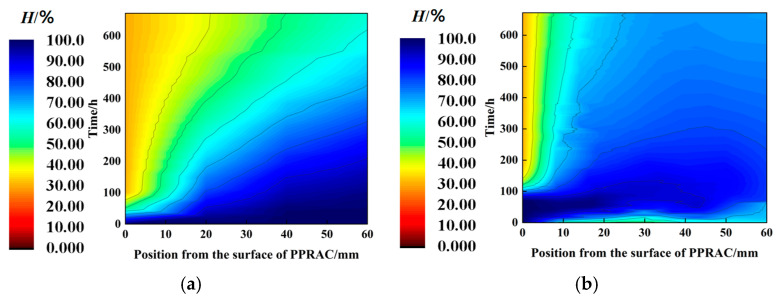
Simulation results of water migration in PPRBAC slab: (**a**) initial pouring stage; (**b**) service period.

This study employs MATLAB-2021a for finite difference calculations to accurately reflect the conditions of the pavement during service and to account for the influence of the wet stage on the dry stage. To quantitatively compare the effects of the wet stage on the dry stage, two scenarios are simulated: external drying and wetting before drying. Most studies assume one-dimensional diffusion in cement-based materials over 28 days under saturated conditions; hence, the simulation duration for both scenarios in this paper is also set to 28 days. The simulation parameters are as follows: during the water absorption phase of the PPRBAC pavement, the relative humidity of the exposed surface is set to 100%, with the wetting process concluding after 70 h. The final moisture content from the wetting phase serves as the initial condition for the drying phase. From 70 h onward, the contact surface is exposed to a relative humidity of 30%, and the analysis concludes 672 h after the start of the wetting phase. For the scenario without considering the wetting process, the initial moisture distribution along the height of the pavement is set to 100%, with identical boundary conditions and analysis time as the drying phase under combined wet–dry conditions. Figure 9 illustrates the calculation results.

**Figure 10:**
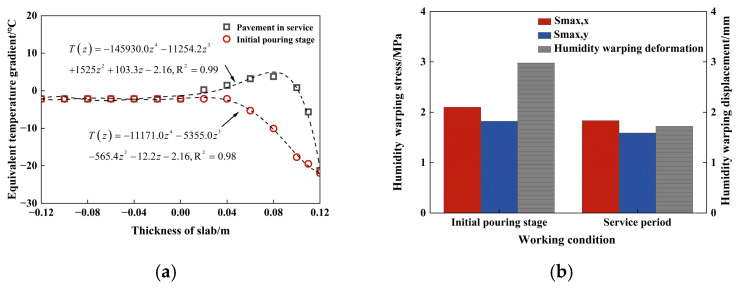
Simulation results of humidity warping of PPRBAC slab: (**a**) equivalent temperature gradient fitting curve; (**b**) comparison of two working conditions.

In the range of 0 to 0.06 m from the exposed surface of PPRBAC, both conditions exhibited a similar trend, with the water loss rate increasing as the distance from the exposed surface decreased. During the service period, the humidity gradient is characterized by higher values in the middle and lower values at the ends. This phenomenon occurs because, during the wetting stage, external wetting conditions have a limited influence on the interior of PPRBAC, resulting in relatively constant relative humidity near the enclosed surface. In contrast, the water loss rate at the surface during the drying stage is significantly higher than that in the middle region. The middle region continues to undergo water diffusion due to the humidity gradient, indicating that it remains in the wetting phase. The impact of these two calculation results on humidity-induced warping stress was investigated to extract and convert data from the 28th day. The conversion results are presented in Figure 10a. A quartic polynomial was also used to fit the results, achieving a fitting accuracy R^2^ exceeding 0.98, which indicates excellent fitting performance.

It is necessary to modify the parameter settings of the finite element model before conducting the finite element analysis, as shown in Figure 7. Road weight considerations are incorporated to ensure that the model accurately reflects actual working conditions, and the contact setting is adjusted to “Hard contact”. At this stage, the equivalent temperature gradient illustrated in Figure 10a is assigned to the model as a predefined field for sequential thermodynamic coupling analysis; the results of this calculation are presented in Figure 10b. The internal humidity gradient distribution of PPRBAC slabs during service is substituted with that from the initial pouring stage. By disregarding the impact of wetting on drying processes, we risk overestimating the moisture warping stress generated by PPRBAC slabs. The final calculated values for warping stress and displacement under this assumption are approximately 1.1 and 1.7 times greater than those derived from an approach where wetting occurs first, followed by drying. Therefore, it is imperative to consider the influence of wet stages on dry stages when calculating moisture-induced warping stress in PPRBAC slabs during service.

### 3.4. Parameter Sensitivity Analysis Based on Taguchi–GRA

This study employs the Taguchi–GRA method for the calculation to investigate further the influence of various factors on PPRBAC moisture warping. Based on the extrapolation results from Section 2.2, it is evident that the warping stress of PPRBAC is predominantly influenced by folding strength, foundation modulus, as well as the length and thickness of the surface layer. Consequently, an orthogonal test table was constructed in this paper, comprising five parameters at four levels each, with the specific parameters for each group detailed in Table 1. The parameters listed in Table 1 are substituted into the model detailed in Section 3.2, and the resulting calculations for maximum humidity warping stress and displacement are presented in Table 2.

**Figure 11:**
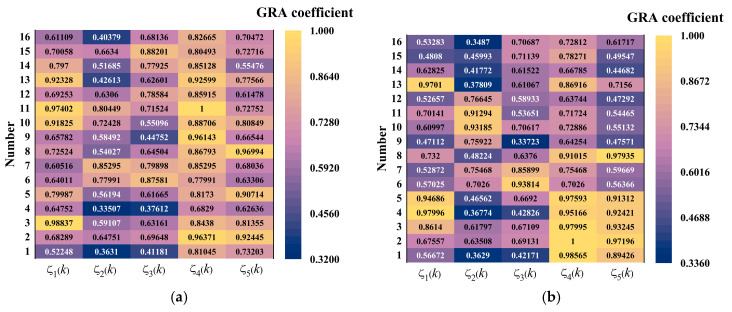
PPRBAC humidity warping gray correlation coefficient: (**a**) humidity warping stress; (**b**) humidity warping displacement.

Equation (37) was utilized to perform dimensionless processing on the data presented in Table 2, while Equation (38) was employed to compute the correlation coefficient. The resultant calculations are illustrated in Figure 11. Based on the gray correlation coefficient analysis of various factors affecting warp stress and humidity-induced displacement, the combination yielding the maximum warp stress is as follows: flexural strength of 5.5 MPa, base modulus of 1000 MPa, subgrade reaction modulus of 100 MPa, slab length of 3.5 m, and slab thickness of 0.22 m. The combination resulting in the maximum warping displacement is characterized by a flexural strength of 5.5 MPa, base modulus of 1500 MPa, subgrade reaction modulus of 125 MPa, slab length of 3.5 m, and slab thickness of 0.20 m. It is evident that the values of warp stress and warping displacement do not reach their maximum when all factors are optimized simultaneously, indicating the presence of a coupling effect among these factors.

**Figure 12:**
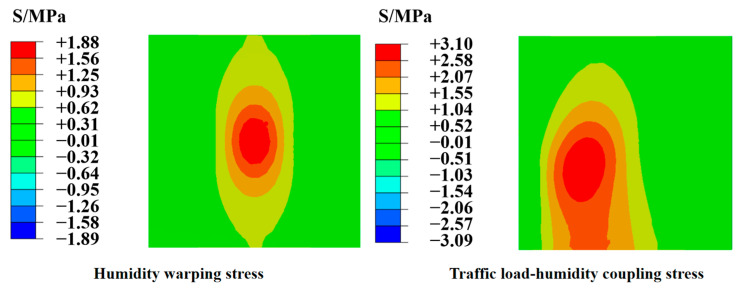
Finite element calculation results for Group 4.

The gray correlation degree of the results presented in Figure 11 was calculated using Formula (39), with the outcomes illustrated in Figure 12. The sensitivity analysis reveals that, in descending order, the factors influencing warping stress are slab length, thickness, flexural strength, foundation reaction modulus, and base modulus. Similarly, the sensitivity ranking for warping displacement is as follows: slab length, flexural strength, thickness, foundation reaction modulus, and base modulus.

PPRBAC pavement, as a kind of rigid pavement, has a large relative stiffness. Although the reaction force distribution at the bottom of the slab is relatively uniform, the change in the reaction modulus of the subgrade and the modulus of the base has a very small effect on the change in the reaction force distribution at the bottom of the slab, and the bending moment generated at the bottom of the slab shows little change. Therefore, compared with other factors, the influence of the substructure on the maximum tensile stress in the pavement slab is limited. Furthermore, as a ribbon structure, PPRBAC pavement exhibits significant sensitivity to environmental changes. The magnitude of its hygroscopic expansion, desiccation contraction, and deformation is directly proportional to the length of the slab. Consequently, longer slabs are more susceptible to humidity-induced warping. An increase in slab thickness enhances the bending stiffness of the section, thereby reducing the likelihood of bending and deformation. Therefore, thicker slabs can better resist humidity-induced warping.

According to the calculation results in Table 2, it can be seen that under normal conditions, humidity warping stress will not cause direct damage to the PPRBAC slab. However, according to the reference [28] (fatigue limit is 0.6), it can be seen that the humidity warping stress of groups 4, 6, 9, 15, and 16 exceeds the fatigue limit. However, the effect of a humidity gradient is prolonged, leading to fatigue cracks under repeated loading. The maximum stress variation in the PPRBAC slab under axial and humidity coupling conditions is primarily determined by the magnitude of warping displacement. Figure 12 shows the finite element calculation results of the humidity-warped stress and the traffic load–humidity coupling stress of the group with the largest warpage displacement (Group 4). It can be seen that when the traffic load is applied to the unfavorable load position (standard axle load with slab angle arrangement of 100 kN), the maximum stress position is 1.65 times that before being considered alone.

**Figure 13:**
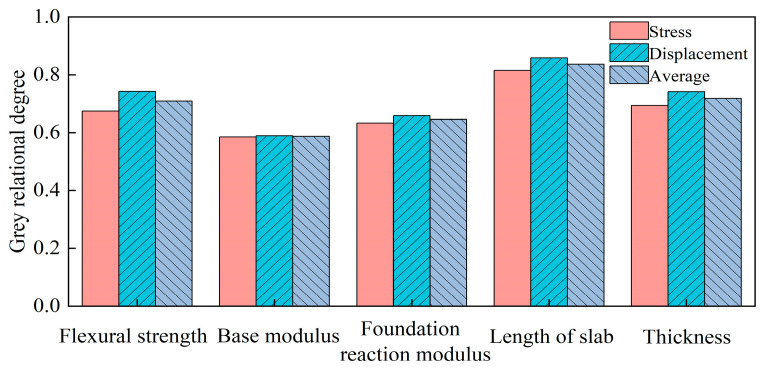
Gray correlation degree of each factor.

Therefore, considering the findings depicted in Figure 13, to mitigate the impact of humidity warping during the calculation and construction of PPRBAC slabs, it is recommended to prioritize slab length, thickness, and flexural strength as key parameters for analysis and control.

## 4. Conclusions

This study investigates the theoretical framework for calculating the moisture warping stress of the PPRBAC slab. Based on prior experimental data, an analytical solution for the humidity warping stress in terms of flexural strength is derived. The methodology for evaluating humidity-induced warping stress during service conditions is also discussed. Furthermore, Taguchi–GRA is employed to conduct a sensitivity analysis of the factors influencing moisture warping, leading to the following conclusions:

(1) In conjunction with prior research findings, the conversion relationship established between flexural strength and elastic modulus demonstrates high accuracy, with a correlation coefficient R^2^ reaching 0.85. The predictive model incorporates adjustments for the BA substitution rate and PPF content, ensuring high reliability.
(2) The nonlinear humidity warping stress formula for PPRBAC pavement slabs is derived based on flexural strength considerations. A comparison between the bending moment equivalent method and the contraction equivalent method reveals that the former fails to accurately capture the nonlinearity of PPRBAC wetness warping stress, leading to a significant underestimation of this stress. Furthermore, numerical simulations validate the rationality and accuracy of the contraction equivalent method.
(3) The PPRBAC pavement slab will experience humidity-induced warping stress and deformation due to the uneven distribution of moisture gradients. The stress state during the service life is calculated based on the assumption of initial pouring conditions (initial saturation, without considering subsequent wetting and drying cycles). This approach tends to overestimate the impact of the wetting phase on the drying phase. Specifically, the final calculated values for warping stress and displacement under this assumption are approximately 1.1 and 1.7 times higher, respectively, compared to the scenario where wetting precedes drying. Therefore, it is essential to account for the influence of the wetting stage on the drying stage when evaluating the humidity-induced warping stress of PPRBAC pavement slabs in service.
(4) The Taguchi–gray relational analysis (Taguchi–GRA) method was employed to determine the weights of each influencing factor associated with PPRBAC road wet-warping. Based on the indices of warping displacement and warping stress, the length, thickness, and flexural strength have a more significant impact on PPRBAC pavement humidity warping than other factors. Therefore, these parameters should be prioritized as key control indices during the design and construction phases.

## Figures and Tables

**Table 1:** Orthogonal experimental design.

Number	Flexural Strength /MPa	Base Modulus /MPa	Foundation Reaction Modulus/MPa	Length of Slab /m	Thickness of Slab/m
1	5.5	2000	125	3.0	0.20
2	5.5	1500	100	3.5	0.22
3	5.5	1000	75	4.0	0.24
4	5.5	500	50	4.5	0.26
5	5.0	2000	75	4.0	0.26
6	5.0	1500	125	4.5	0.24
7	5.0	1000	50	3.0	0.22
8	5.0	500	100	3.5	0.20
9	4.5	2000	50	4.5	0.22
10	4.5	1500	125	4.0	0.20
11	4.5	1000	100	3.5	0.26
12	4.5	500	75	3.0	0.24
13	4.0	2000	50	3.5	0.24
14	4.0	1500	75	3.0	0.26
15	4.0	1000	100	4.5	0.20
16	4.0	500	125	4.0	0.22

**Table 2:** The results of Taguchi orthogonal experimental.

Number	Flexural Strength /MPa	Base Modulus /MPa	Foundation Reaction Modulus/MPa	Length of Slab /m	Thickness of Slab/m	Warping Stress/MPa	Warping Displacement/mm
1	5.5	2000	125	3.0	0.20	1.299	1.667
2	5.5	1500	100	3.5	0.22	1.502	2.252
3	5.5	1000	75	4.0	0.24	1.730	2.885
4	5.5	500	50	4.5	0.26	1.876	3.578
5	5.0	2000	75	4.0	0.26	1.733	2.935
6	5.0	1500	125	4.5	0.24	2.231	3.341
7	5.0	1000	50	3.0	0.22	1.040	1.747
8	5.0	500	100	3.5	0.20	1.415	2.103
9	4.5	2000	50	4.5	0.22	2.326	3.026
10	4.5	1500	125	4.0	0.20	1.981	2.465
11	4.5	1000	100	3.5	0.26	1.204	2.301
12	4.5	500	75	3.0	0.24	0.866	1.758
13	4.0	2000	50	3.5	0.24	1.378	2.197
14	4.0	1500	75	3.0	0.26	0.917	1.744
15	4.0	1000	100	4.5	0.20	2.127	2.645
16	4.0	500	125	4.0	0.22	1.982	2.917

## Data Availability

The datasets used and/or analyzed during the current study are available from the corresponding author on reasonable request.
